# Laparoscopic management of small bowel obstruction due to unusual foreign body ingestion in a child with autism: a case report

**DOI:** 10.1093/jscr/rjaf615

**Published:** 2025-09-05

**Authors:** Nasir M Bustangi, Bashair Al-Bihani

**Affiliations:** Division of Pediatric Surgery, Department of Surgery, Faculty of Medicine, King Abdulaziz University Hospital, King Abdulaziz University, Almortada 7441, Jeddah 22252, Saudi Arabia; Department of Pediatric Surgery, International Medical Center, Hail Street, AL-Ruwais, Jeddah 23214, Saudi Arabia

**Keywords:** foreign body ingestion, small bowel obstruction, laparoscopy, pediatric surgery, autism

## Abstract

Foreign body ingestion in children, especially those aged 6 months to 3 years, is a common clinical concern. While most objects pass through the gastrointestinal tract uneventfully, some may result in obstruction and necessitate surgical intervention. We report a rare case of a 10-year-old child with autism who presented with small bowel obstruction following ingestion of a rubber feeding bottle nipple. Imaging confirmed the object lodged in the proximal jejunum. The patient underwent successful laparoscopic extraction of the foreign body and concurrent appendectomy for acute appendicitis. Postoperative recovery was uneventful, and the patient was discharged on Day 5. This case supports laparoscopic intervention as a safe and effective option for managing gastrointestinal foreign bodies and concomitant intra-abdominal pathology in pediatric patients.

## Introduction

Foreign body ingestion is a frequent occurrence in pediatric practice, peaking in children between 6 months and 3 years of age. In older children, ingestion may be associated with behavioral conditions, such as autism or other neurodevelopmental disorders [1]. While most foreign bodies pass spontaneously, a subset requires surgical intervention due to impaction or complications [1].

Laparoscopic surgery has emerged as a minimally invasive alternative to open procedures, offering reduced postoperative pain, faster recovery, and better cosmetic outcomes [1]. Here, we present a case highlighting the feasibility and benefits of laparoscopic management in a pediatric patient with autism presenting with a rare jejunal foreign body and concurrent appendicitis.

## Case presentation

A 10-year-old boy with known autism (not on pharmacologic therapy) presented with a 4-day history of vomiting, abdominal pain, and poor oral intake following ingestion of a rubber feeding bottle nipple. A similar incident 2 years prior was managed endoscopically. Examination revealed signs of dehydration and localized abdominal tenderness, particularly in the right iliac fossa. Vital signs showed tachycardia (122 bpm).

**Figure 1:**
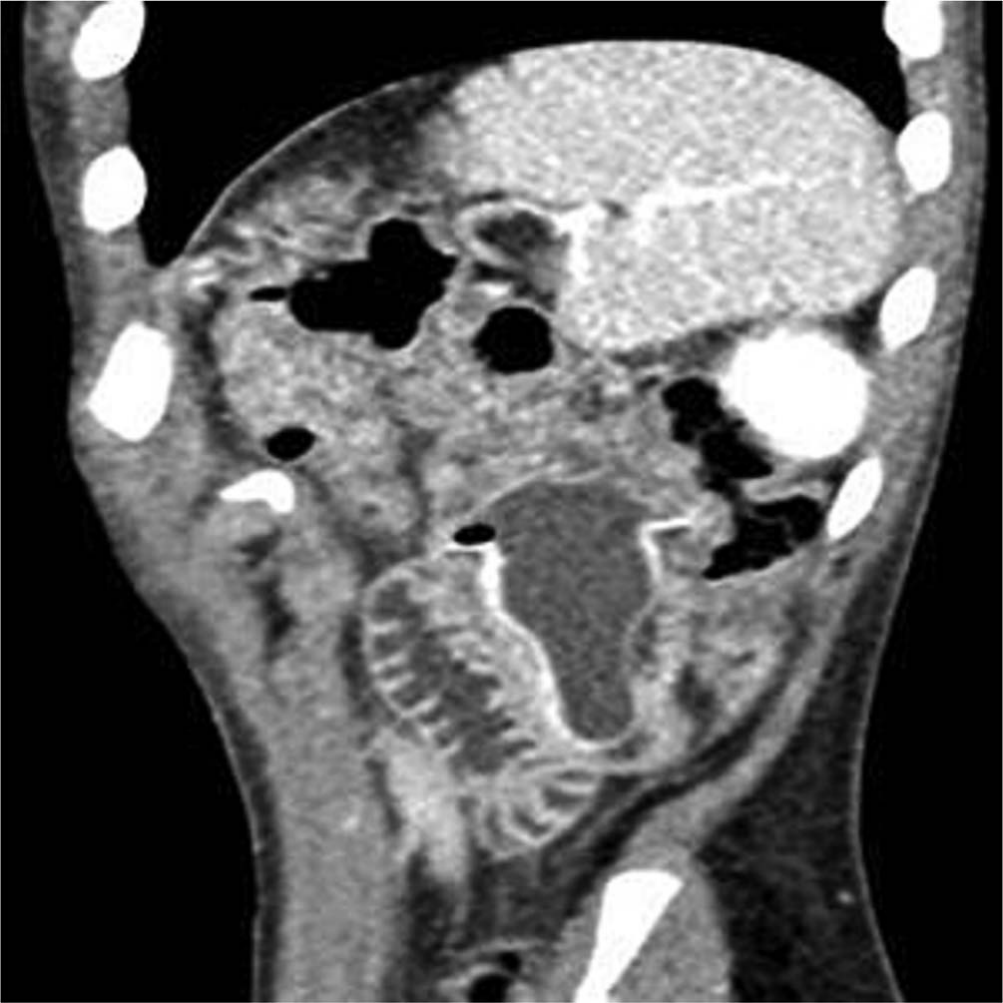
CT sagittal view showing fluid-filled nipple of feeding bottle lodged in jejunum.

Imaging and laboratory findings:

- Abdominal X-ray: Dilated small bowel loops with multiple air-fluid levels.
- Laboratory tests: WBC count 12 100/μl, potassium 3 mEq/L.
- CT abdomen with contrast: Foreign body in the proximal jejunum with partial obstruction, dilated appendix (9 mm) with wall enhancement and fat stranding consistent with acute appendicitis (Fig. 1).

## Treatment and outcome

**Figure 2:**
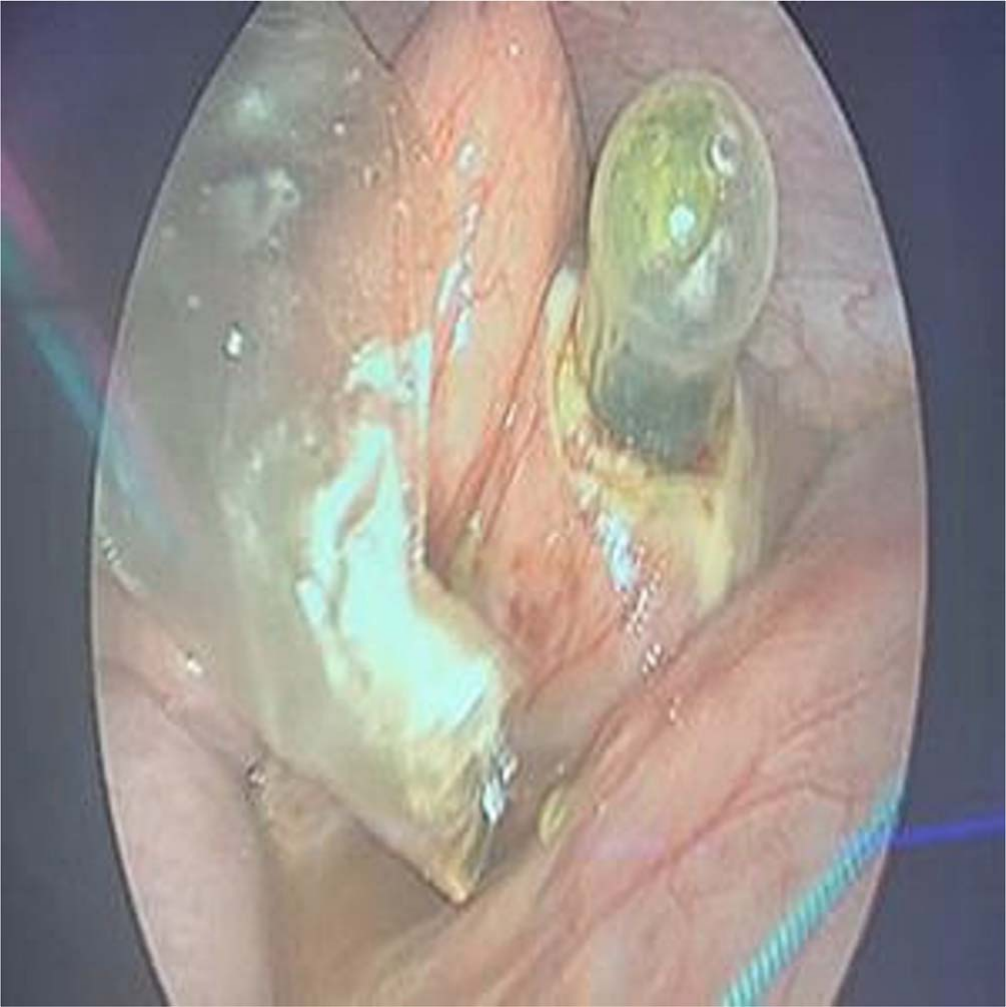
Extracted rubber nipple post-enterotomy.

**Figure 3:**
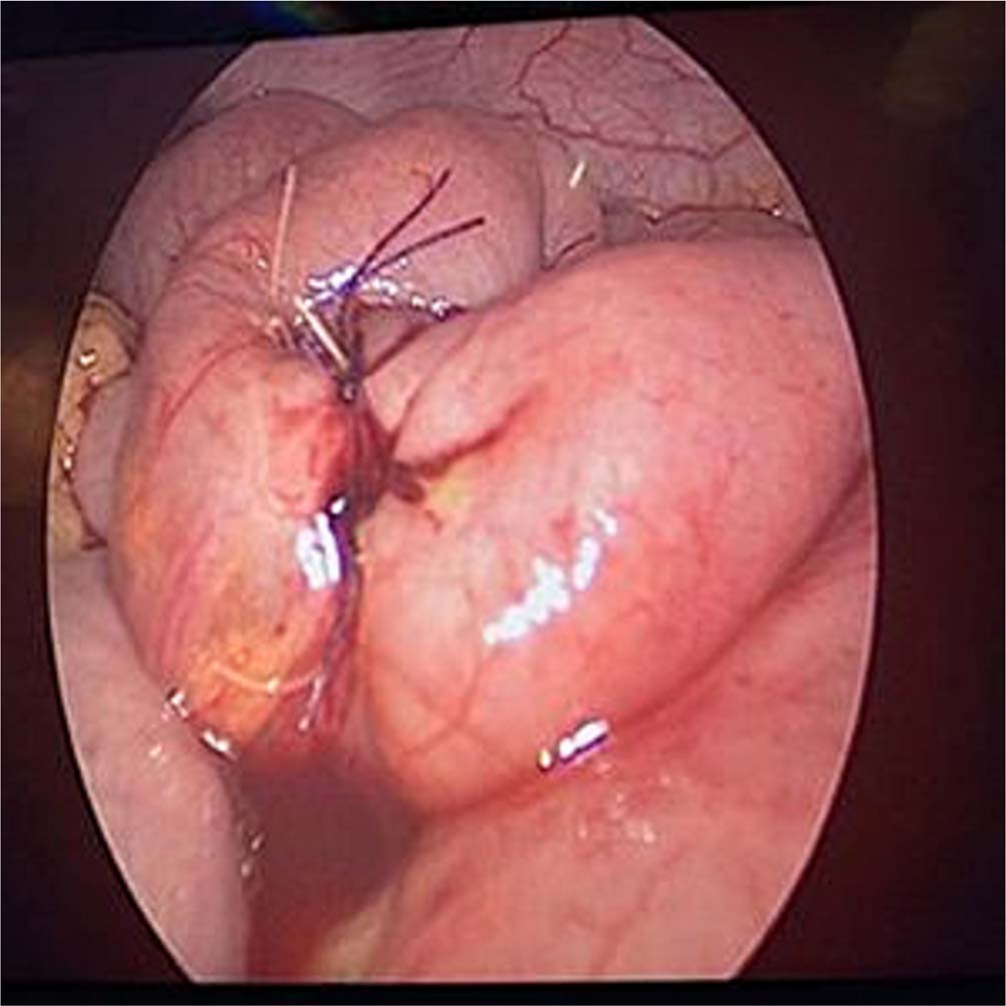
Closure of enterotomy site following foreign body removal.

**Figure 4:**
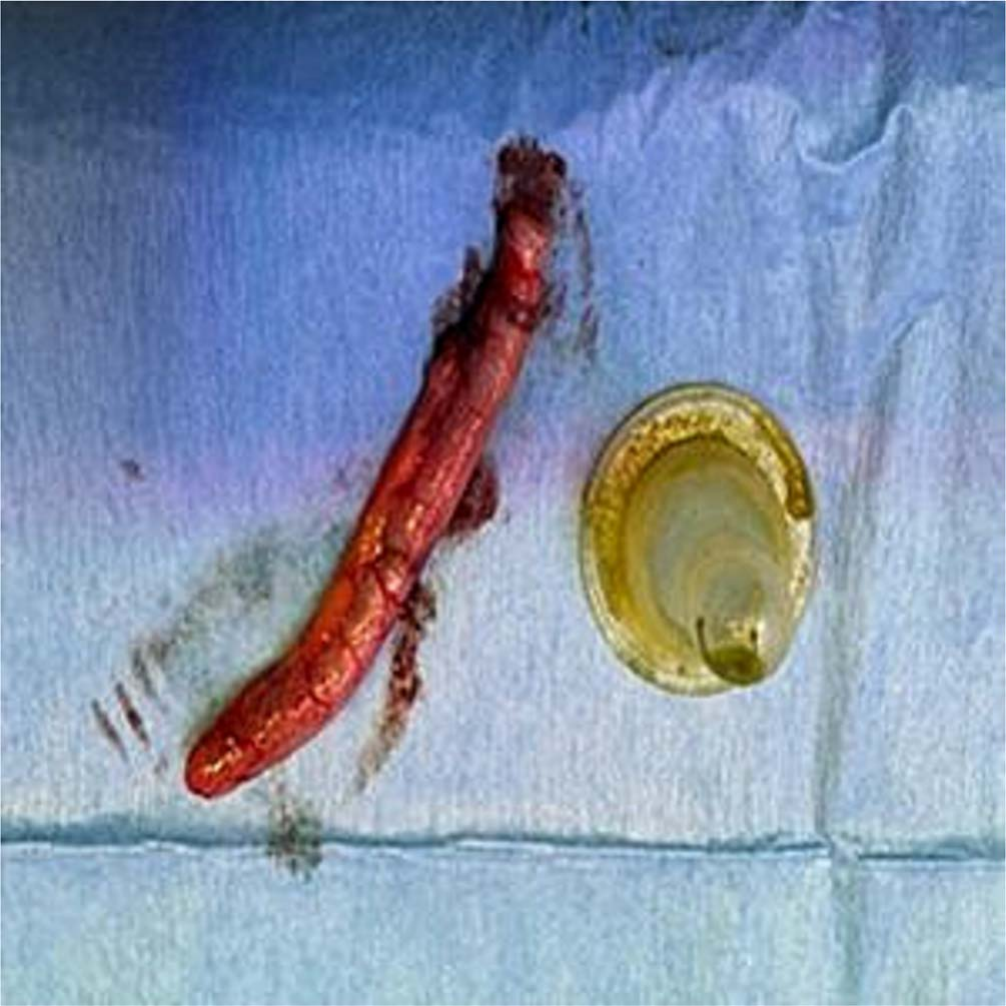
Resected appendix and retrieved foreign body.

The patient was stabilized with IV fluids, nasogastric decompression, and antibiotics. Emergency laparoscopic surgery was performed using a trans-umbilical Hasson technique for camera access and two 5 mm working ports. Intraoperative findings included a foreign body lodged in the proximal jejunum without adhesions. Transparietal suspension sutures were used to facilitate exposure. Enterotomy was performed, and the object was extracted via an endo-bag. The enterotomy site was closed using STRATAFIX™ 3.0 suture (Figs 2 and 3). A laparoscopic appendectomy was concurrently performed via the umbilical incision using the in-out technique (Fig. 4).

Postoperatively, the patient tolerated oral intake within 8 hr and was discharged on Day 5. Psychiatry consultation led to initiation of risperidone. Histopathology of the appendix confirmed acute appendicitis with *Enterobius vermicularis* infestation, for which the patient was started on mebendazole after infectious disease consultation.

## Discussion

Although most foreign body ingestions resolve spontaneously, approximately 10%–20% require endoscopic or surgical intervention, and fewer than 1% necessitate open surgery [2]. Unusual objects, such as rubber components, can cause partial or complete obstruction, particularly when lodged in the jejunum.

Laparoscopy has been increasingly utilized for both diagnosis and treatment of gastrointestinal emergencies in children. It offers the benefit of targeted exploration, reduced morbidity, and faster return to normal activities [3–5]. Our use of transparietal suspension sutures minimized spillage risk and improved exposure during enterotomy. Concurrent appendectomy in the same session demonstrated the flexibility and diagnostic utility of laparoscopic surgery.

While experience and access to equipment may limit its widespread use in all centers, cases like this emphasize its utility even in emergency settings and patients with complex behavioral conditions like autism.

## Conclusion

Laparoscopy is a viable and safe option for managing small bowel obstructions due to foreign body ingestion in pediatric patients. It also permits simultaneous treatment of incidental findings, such as appendicitis. Broader adoption of this approach may improve outcomes and reduce recovery time in appropriately selected patients.
